# Cross-Modal Scene Prior for Adaptive RGB-Guided Infrared Column Stripe Noise Removal

**DOI:** 10.3390/s26123638

**Published:** 2026-06-07

**Authors:** Bahri Abaci, Seniha Esen Yuksel

**Affiliations:** 1 Department of Electrical and Electronics Engineering, Hacettepe University, Ankara 06800, Turkey; eyuksel@ee.hacettepe.edu.tr; 2 ASELSAN Inc., Ankara 06830, Turkey

**Keywords:** infrared imaging, non-uniformity correction, column stripe noise, cross-modal guidance, spatially adaptive normalization, deep learning, visible-light guidance

## Abstract

Infrared focal plane array detectors produce column stripe noise due to inter-detector response variations. Existing single-frame correction methods operate exclusively on the degraded infrared image and cannot reliably distinguish column noise from genuine vertical scene structures. With the increasing availability of co-registered visible-light cameras in modern electro-optical/infrared payloads, we propose to exploit the visible image as a structural guide for infrared destriping. Through a cross-modal correlation analysis, we show that the structural correspondence between RGB and infrared images is spatially non-uniform, motivating a selective rather than uniform fusion strategy. Based on this observation, we propose CMSP (Cross-Modal Scene Prior), a lightweight single-frame denoising architecture that selectively applies RGB guidance where it is beneficial. The proposed AdaptiveSPADE module blends RGB-guided modulation with standard instance normalization through a learned per-pixel confidence map, while a dual-path output head separately estimates pixel-wise residuals and column-constant stripe patterns. Evaluated on three public RGB–IR datasets, CMSP achieves 51.91 dB PSNR on M3FD, outperforming the best baseline by 5.79 dB with only 638 K parameters. A downstream evaluation on real stripe noise demonstrates that CMSP not only removes artifacts but also preserves the fine structures critical for infrared small target detection. Ablation studies confirm that adaptive gating more than doubles the benefit of RGB guidance compared to uniform modulation, and prevents degradation when cross-modal alignment is weak.

## 1. Introduction

Infrared imaging systems rely on two-dimensional focal plane arrays where each detector element converts incident thermal radiation into an electrical signal [1]. Due to manufacturing tolerances and variations in readout circuitry, the individual response of each detector differs in both gain and offset, producing spatially fixed artifacts known as column stripe noise. These artifacts, particularly the column-wise additive offset component, degrade image quality and impair downstream tasks such as target detection, tracking, and autonomous navigation. Standard calibration procedures correct for these non-uniformities by periodically closing a mechanical shutter to re-estimate offset values [2,3]. However, shutter-based correction interrupts image acquisition and fails to address lens-induced artifacts. This limitation has motivated the development of scene-based and learning-based non-uniformity correction (NUC) methods that operate directly on captured imagery [4,5,6,7].

Classical single-frame techniques employ directional filtering [8] or weighted least squares estimation [5] to separate stripe patterns from scene content. More recently, deep learning methods have achieved notable improvements by learning the mapping between noisy and clean infrared images through convolutional architectures [4], wavelet-domain representations [7], and unsupervised generative frameworks [6]. While effective, all of these methods rely exclusively on the information available within the degraded infrared image itself. This single-modal constraint limits their ability to distinguish column noise from genuine vertical scene structures, as both exhibit similar spatial characteristics in the infrared domain.

Modern electro-optical and infrared (EO/IR) payloads routinely integrate co-registered visible-light cameras alongside thermal sensors [9]. The visible-light image captures the same scene geometry but is inherently free of the column-wise fixed-pattern noise that affects the infrared channel. This complementary modality represents a potential source of structural guidance for the denoising process. Recent works have begun to explore this direction: Mou et al. [10] proposed a GAN-based framework that uses visible images to assist infrared NUC through channel-level attention, and our earlier work [11] demonstrated the benefit of RGB guidance in a multi-frame setting. In this paper, we investigate the spatially varying nature of this cross-modal relationship and propose an adaptive mechanism that selectively applies RGB guidance where it is most effective.

Visible and thermal images capture different physical phenomena (reflected light versus emitted radiation), which raises a question about whether any meaningful correlation exists between the two modalities at the spatial resolution relevant to column noise correction. To address this, we conduct a blockwise cross-modal correlation analysis between the horizontal discontinuities of RGB and clean infrared images. The analysis reveals that the block-level correlation is spatially non-uniform, with approximately 12% of 32×32 image blocks exhibiting absolute correlation above 0.3, while a large fraction shows negligible agreement. This finding motivates a selective rather than uniform fusion strategy. Based on this observation, we propose CMSP (Cross-Modal Scene Prior), a single-frame denoising architecture that incorporates AdaptiveSPADE, a normalization module that blends RGB-guided modulation with standard instance normalization through a learned per-pixel confidence map. The backbone employs stripe-aware depthwise convolutions, and a dual-path output head separately estimates pixel-wise residuals and column-constant stripe patterns.

The proposed method is evaluated on three public datasets containing co-registered RGB–IR image pairs: M3FD [12], MSRS [13], and the Lynred Mobility Dataset [14]. Experimental results show that CMSP outperforms recent destriping methods, including D1WLS [5], DLS-NUC [4], DestripeCycleGAN (DCGAN) [6], and ASCNet [7], across all three datasets, and a downstream evaluation on real stripe noise demonstrates that CMSP improves infrared small target detection while maintaining low false-alarm rates. Ablation studies confirm that the adaptive gating mechanism more than doubles the benefit of RGB guidance compared to uniform SPADE modulation.

The main contributions of this work are as follows:

- A cross-modal correlation analysis demonstrating that the structural agreement between visible and infrared column discontinuities is spatially non-uniform, providing empirical motivation for adaptive fusion.
- An adaptive cross-modal gating module (AdaptiveSPADE) with noise-resilient confidence estimation based on column-mean subtraction, which selectively applies RGB guidance at spatial locations where it is beneficial.
- A lightweight single-frame denoising architecture evaluated on three public RGB–IR datasets, achieving state-of-the-art performance in column stripe noise removal.

The remainder of this paper is organized as follows. Section 2 reviews related work on infrared destriping and cross-modal image restoration. Section 3 presents the proposed method, including the correlation analysis and the AdaptiveSPADE module. Section 4 describes the experimental setup and reports quantitative and qualitative results. Section 5 discusses the findings and their implications. Section 6 concludes the paper.

## 2. Related Work

The proposed method builds on two research directions: single-frame infrared destriping, which addresses column noise using only the degraded infrared image; and cross-modal guided restoration, which leverages a secondary modality to improve the quality of a target image. Below, we review relevant work in each area and identify the gaps that motivate our approach.

### 2.1. Single-Frame Infrared Destriping

Column stripe noise removal from infrared images has been addressed through both classical and learning-based approaches. Classical methods typically exploit the directional structure of stripe noise through filtering or optimization. Cao et al. [8] employed sequential one-dimensional filters, applying horizontal edge-preserving filtering followed by vertical separation to isolate stripe components from scene texture. Li et al. [5] proposed D1WLS, which combines horizontal weighted least squares filtering with vertical ridge regression to correct column-wise offsets while preserving edge content.

Learning-based methods have achieved stronger results by training on synthetic noise–image pairs. He et al. [4] introduced DLS-NUC, a residual convolutional network that directly estimates fixed-pattern noise from a single infrared frame. Saragadam et al. [15] combined a Deep Image Prior framework with a physical sensor model to jointly decompose additive and multiplicative noise components from a short sequence of jittered frames. Liu et al. [16] extended this approach with a total variation regularizer whose gradient neighborhood adapts during optimization. Li et al. [17] addressed both stripe and low-frequency shading through wavelet–domain noise separation, combining clustering-based stripe extraction in the vertical wavelet components with Bezier surface fitting for low-frequency artifacts. Yang et al. [6] proposed DestripeCycleGAN, an unsupervised framework that integrates a stripe generation model with a Haar wavelet background guidance module within a CycleGAN architecture, eliminating the need for paired training data. Most recently, Yuan et al. [7] introduced ASCNet, which replaces the conventional inverse wavelet upsampler with pixel shuffle to avoid semantic bias, and incorporates a column non-uniformity correction module that captures long-range column dependencies through spatial and self-calibrated attention.

All of the above methods operate exclusively on infrared data. While they effectively suppress stripe artifacts in many scenarios, their single-modal nature limits the ability to distinguish column noise from true vertical scene structures, particularly in images with strong vertical edges or low-texture regions where stripe patterns and scene content overlap spatially.

### 2.2. Cross-Modal Guided Image Restoration

Guided image restoration leverages structural or contextual information from a secondary modality to improve the quality of a primary image. Wang et al. [18] used near-infrared images to guide visible-spectrum denoising by concatenating the two modalities into a four-channel input processed by a UNet architecture. Sheng et al. [19] addressed cross-spectral stereo denoising, accounting for pixel-level disparities between left and right views during NIR-guided RGB restoration. In the thermal-to-visible direction, Cao et al. [20] proposed TGLLE-Net, which uses thermal images to guide low-light visible image enhancement through stacked guided convolutions.

In the specific context of infrared non-uniformity correction, Mou et al. [10] proposed VIA-NUC, a dual-discriminator GAN that uses co-registered visible images to guide infrared NUC. The generator processes the two modalities through separate encoding paths and fuses them via SEBlock channel attention during decoding. Our earlier work [11] demonstrated that RGB guidance improves infrared NUC accuracy in a multi-frame setting through input-level concatenation.

While these methods establish that a visible image can guide infrared restoration, none of them varies the strength of the fusion across spatial locations: Wang et al. [18] and our earlier work [11] concatenate the modalities at the input, Sheng et al. [19] and Cao et al. [20] fuse through convolutional guidance, and VIA-NUC [10] reweights spliced infrared–visible features with an SEBlock that rescales each channel uniformly over all spatial positions. CMSP instead introduces AdaptiveSPADE, which explicitly weights the RGB contribution per pixel according to the learned cross-modal correlation between infrared and visible structure (Section 3.2), applying guidance only where the two modalities agree and reverting to instance normalization elsewhere. The synthetic stripe noise we train on is physically grounded: the per-column gain and offset are drawn as $\alpha_{j}∼U\left(0.985,1.015\right)$ and $\beta_{j}∼N\left(0,0.015\right)$ following the real focal-plane-array sensor characterization of [21], so the contribution of CMSP lies in the adaptive fusion rather than in a more aggressive noise model. In terms of deployment, CMSP is a single, non-adversarial network of only 638 K parameters with publicly released code, in contrast to the adversarial dual-discriminator framework of VIA-NUC. Finally, CMSP is trained on a single dataset (M3FD) and evaluated on two further datasets (MSRS and Lynred) without retraining, directly assessing cross-dataset generalization; VIA-NUC, by contrast, reports results on held-out splits of its own training data.

## 3. Methodology

### 3.1. Problem Formulation

Let $z\in R^{H\times W}$ denote a noisy infrared image acquired by a focal plane array detector, and let $x\in R^{H\times W}$ denote the corresponding clean scene content. Following the linear non-uniformity model widely adopted in the literature [21], the clean image can be recovered as:

(1) $$x_{ij}=\alpha_{ij}\;z_{ij}+\beta_{ij},$$

where $\alpha_{ij}$ and $\beta_{ij}$ are the gain and offset parameters at pixel (i,j). Because column stripe noise stems from the column-parallel readout architecture, $\alpha_{ij}$ and $\beta_{ij}$ are typically assumed to be constant along each column.

Recovering both parameters from a single observation is ill-posed, since Equation (1) constitutes a single equation with two unknowns per pixel. Multi-frame methods resolve this ambiguity by observing multiple scenes under the same noise conditions, producing independent equations that allow the gain and offset to be separated [11]. In the single-frame setting, no such redundancy is available.

In this work, we address the single-frame setting by restricting the problem to estimate the offset component β. This restriction is supported by real sensor characterization [21], which shows that the gain values fall within a narrow range ($\alpha_{ij}\in \left[0.985,1.015\right]$). Even so, the multiplicative interaction between $\alpha_{ij}$ and the scene content $x_{ij}$ means that the effective per-pixel offset the network must estimate is not column-constant but varies with scene intensity. The denoising task therefore reduces to estimating a per-pixel offset map $\beta \in R^{H\times W}$ that contains both a dominant column-structured component and a scene-dependent pixel-wise residual. The challenge lies in distinguishing these structured offsets from genuine vertical scene content, which may produce similar inter-column discontinuities.

### 3.2. Cross-Modal Correlation Analysis

To investigate whether co-registered RGB images carry information relevant to column stripe noise removal, we analyze the structural correlation between horizontal discontinuities of RGB and clean infrared images. Specifically, for each image pair, we compute horizontal finite differences $\Delta_{i,j}^{\mathrm{RGB}}=g_{i,j+1}-g_{i,j}$ and $\Delta_{i,j}^{\mathrm{IR}}=x_{i,j+1}-x_{i,j}$, where *g* denotes the grayscale-converted RGB image. These differences capture the inter-column transitions that are directly affected by column stripe noise.

We divide each difference map into non-overlapping blocks of size B×B and compute the absolute Pearson correlation |ρ| between the RGB and IR blocks at each spatial position. We use the Pearson correlation of horizontal differences as a simple descriptor of cross-modal structural agreement; the direction is not critical, as vertical differences yield comparable correlations. The absolute value is used because a learned network can exploit both positive and negative correlations equally. The analysis is conducted over the entire M3FD dataset (4200 image pairs) at three block sizes.

The results in Table 1 reveal that the blockwise correlation is consistently above the random baseline across all block sizes, confirming the presence of genuine cross-modal structural correspondence. At the same time, the distribution is non-uniform: a notable fraction of blocks exhibit strong correlation (suitable for RGB-guided correction), while a comparable fraction shows negligible correlation (where RGB guidance would not contribute). This non-uniformity motivates an adaptive fusion mechanism that concentrates RGB guidance on regions where it is informative, rather than applying it uniformly across the entire image.

### 3.3. Network Architecture

**Figure 1:**
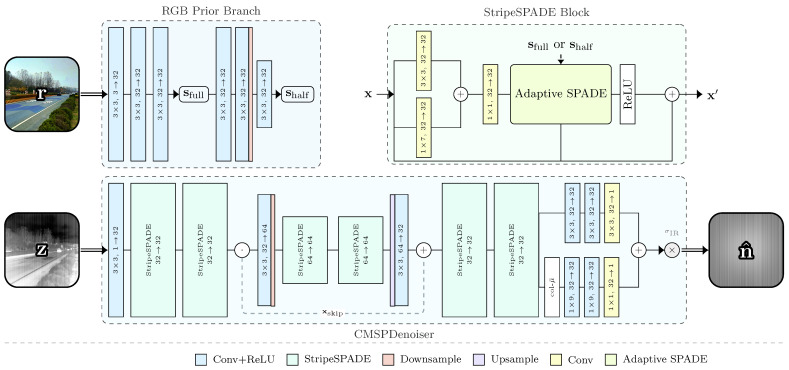
Overview of the proposed CMSP architecture. The network processes a noisy infrared image through a stripe-aware encoder–decoder backbone. RGB guidance is injected via AdaptiveSPADE modules that selectively modulate infrared features based on a learned confidence map. The dual-path output head estimates pixel-wise residuals and column-constant stripe patterns separately.

The proposed architecture, illustrated in Figure 1, is a single-frame encoder–decoder network that processes a noisy infrared image and optionally incorporates guidance from a co-registered RGB image. The RGB prior branch extracts multi-scale structural features from the visible-light image, which are injected into the backbone through StripeSPADE blocks. Each StripeSPADE block combines stripe-aware depthwise convolutions with AdaptiveSPADE modulation, and these blocks form the building units of the encoder–decoder backbone. The backbone terminates in a dual-path output head that separately estimates pixel-wise residuals and column-constant stripe patterns. The following subsections describe each component in detail.

#### 3.3.1. RGB Prior Branch

When RGB guidance is available, a lightweight convolutional branch extracts multi-scale structural priors from the visible-light image $r\in R^{3\times H\times W}$. The branch consists of three 3×3 convolutional layers with ReLU activations, producing a feature map $s_{\mathrm{full}}\in R^{C_{p}\times H\times W}$ at full resolution. A further three convolutional layers (with stride-2 downsampling in the middle) are applied to $s_{\mathrm{full}}$ to yield the half-resolution prior $s_{\mathrm{half}}\in R^{C_{p}\times H/2\times W/2}$ used in the bottleneck. Here, $C_{p}=32$ is the prior channel dimension. In the ablation setting without RGB guidance, both priors are omitted and the AdaptiveSPADE modules fall back to standard instance normalization.

#### 3.3.2. Stripe-Aware Encoder–Decoder

The noisy infrared input z is first normalized to zero mean and unit variance using its spatial mean $\mu_{z}$ and standard deviation $\sigma_{z}$. The standard deviation $\sigma_{z}$ is retained and later used to rescale the network output back to the original intensity range. This normalization decouples the backbone from scene-dependent intensity variations and is essential for cross-dataset generalization, since different sensors and scenes produce different noise magnitudes.

The normalized input is projected into a 32-channel feature map by a 3×3 convolution, followed by a ReLU activation. The backbone is then organized as a three-level architecture with two encoder blocks at full resolution, two bottleneck blocks at half resolution, and two decoder blocks at full resolution. Each block is a StripeSPADE block consisting of two parallel depthwise convolutions, a 3×3 kernel that captures local spatial patterns and a 1×7 horizontally elongated kernel that aligns with the directional structure of column stripe noise. The outputs of both branches are summed, mixed through a 1×1 pointwise convolution, passed through AdaptiveSPADE modulation and an ReLU activation, and finally combined with the block input through a residual connection, as shown in Figure 1. Downsampling between the encoder and bottleneck is performed via a stride-2 convolution, and upsampling is performed via bilinear interpolation followed by a 3×3 convolution. A skip connection links the encoder output to the decoder input.

#### 3.3.3. Dual-Path Output Head

The decoder output is processed by two parallel branches. The residual branch applies two 3×3 convolutional layers with ReLU activations followed by a 3×3 convolution to produce a pixel-wise residual map $r_{\mathrm{px}}\in R^{1\times H\times W}$. The stripe branch averages the decoder features along the row dimension, applies two 1×9 horizontal convolutions with ReLU activations, and projects to a single channel through a 1×1 convolution. The resulting column vector is broadcast to the full spatial size to produce $r_{\mathrm{col}}\in R^{1\times H\times W}$. This branch explicitly models the column-constant β component of the offset, consistent with the column-parallel readout origin of stripe noise. The residual branch complements it by estimating the per-pixel correction that arises from the multiplicative interaction of $\alpha_{ij}$ with the scene content. The two components are summed, scaled by $\sigma_{z}$, and subtracted from the noisy input to obtain the denoised image:

(2) $$\hat{x}=z-\left(r_{\mathrm{px}}+r_{\mathrm{col}}\right)\cdot \sigma_{z}.$$

### 3.4. Adaptive Cross-Modal Gating

Spatially adaptive denormalization (SPADE) [22] injects external guidance into a feature map by modulating instance-normalized features with spatially varying affine parameters derived from a conditioning input. For a feature tensor x and an RGB prior s, the operation is:

(3) SPADE(x,s)=γ(s)⊙IN(x)+β(s),

where IN(·) denotes instance normalization, and γ(s), β(s) are learned from s through a shared convolutional layer followed by separate 3×3 convolutions. This modulation is applied identically at every spatial location, regardless of whether the RGB prior carries useful structural information at that position.

As established in Section 3.2, the cross-modal correlation between RGB and IR is spatially non-uniform. To exploit this structure, we propose AdaptiveSPADE, which introduces a per-pixel confidence map $c\in {\left[0,1\right]}^{C\times H\times W}$ that gates the blend between SPADE-modulated and unmodulated features:

(4) AdaptiveSPADE(x,s)=c⊙SPADE(x,s)+(1−c)⊙IN(x).

where c approaches 1, the network applies full RGB-guided modulation; and where c approaches 0, it relies on instance normalization alone. The confidence map is estimated as $c=\sigma (G\left(\left[\tilde{x};\;s\right]\right))$, where σ is the sigmoid activation, [·;·] denotes channel-wise concatenation of the column-mean-subtracted infrared features $\tilde{x}$ and the RGB prior s, and *G* consists of two 3×3 convolutional layers with an ReLU activation in between.

A critical consideration is that the input features x are contaminated by the very column stripe noise the network aims to remove. If the raw features were used to estimate c, the gate would conflate noise-induced inter-column discontinuities with genuine cross-modal disagreement. To address this, we subtract the per-column mean from the feature map before feeding it to the gating network. Because the dominant column-structured component of the noise contributes equally to all rows of a given column, it largely cancels in the subtraction, leaving a representation that reflects primarily the within-column variation of the scene content. This column-mean-subtracted feature is used as the infrared input to the gating network, making the confidence estimation robust to column stripe noise.

When no RGB input is provided (s = None), the AdaptiveSPADE module reduces to standard instance normalization, allowing the architecture to function as a purely infrared denoiser without modification.

### 3.5. Training Strategy

The network is trained on the M3FD dataset [12], which contains 4200 co-registered RGB–IR image pairs captured in diverse outdoor scenarios. The dataset is partitioned into training and testing subsets at a 70:30 ratio, yielding 2940 pairs for training and 1260 for testing. Cross-dataset generalization is evaluated on the MSRS [13] and Lynred Mobility [14] datasets without any retraining.

To generate training pairs, synthetic column stripe noise is injected into the clean infrared images. Each stripe pattern is defined by per-column gain and offset parameters sampled as $\alpha_{j}∼U\left(0.985,1.015\right)$ and $\beta_{j}∼N\left(0,0.015\right)$, following the sensor characterization reported in [21]. To check that this range is realistic, we estimated the per-column offset on real stripe-contaminated IRSTDID-800 images, using destriped outputs as an approximate clean reference. The estimated offset standard deviation is 0.023 of the normalized intensity range, compared with 0.015 in the synthetic model, indicating a comparable noise magnitude. As different sensors may exhibit different offset ranges, a systematic study across noise levels is left as future work. A total of 10,000 noise patterns are generated, with 9000 used for training and 1000 reserved for testing. Each training sample pairs a randomly selected scene with a randomly selected noise pattern.

Training images are resized to 320×288 and randomly cropped to 288×288. Horizontal flipping is applied for data augmentation. The network is trained for 80 epochs using the AdamW optimizer with a learning rate of $1\times 10^{-4}$ and a batch size of 8. The loss function is the $L_{1}$ distance between the predicted ($\hat{x}$) and ground truth clean infrared images (x). We use a simple $L_{1}$ objective because the column-wise structure of the noise is already addressed architecturally by the dedicated column-constant branch of the dual-path output head, rather than imposed through the loss. A frequency-domain or column-wise regularization term is a reasonable extension that could further suppress residual stripes, which we leave as future work.

## 4. Experimental Results

### 4.1. Datasets

The proposed method is evaluated on three public datasets containing co-registered RGB–IR image pairs.

**M3FD** [12] contains 4200 aligned RGB–IR pairs captured in diverse outdoor conditions including daytime, nighttime, fog, and heavy traffic. Images have a spatial resolution of 1024×768. Following the split described in Section 3.5, 2940 pairs are used for training and 1260 for testing.

**MSRS** [13] provides 1444 co-registered RGB–IR pairs at 640×480 resolution, collected in daytime and nighttime road scenarios. The full dataset is used exclusively for testing to evaluate cross-dataset generalization.

**Lynred Mobility** [14] is a detection-oriented dataset containing 4000 aligned RGB–IR pairs. The dataset is used exclusively for testing, without any retraining.

For all three datasets, testing uses 1000 synthetic noise patterns reserved from the noise generation described in Section 3.5. Each noise pattern is paired with a randomly sampled image from the test split of the respective dataset, producing 1000 noise–image pairs per dataset, and the metrics are averaged over these pairs.

### 4.2. Evaluation Metrics

Three standard image quality metrics are used for evaluation. Peak Signal-to-Noise Ratio (PSNR) [23] measures overall reconstruction accuracy. Structural Similarity Index (SSIM) [24] captures luminance, contrast, and structural consistency. Gradient Magnitude Similarity Deviation (GMSD) [25] quantifies texture and edge preservation through gradient magnitude comparison. Higher PSNR and SSIM values indicate better quality, while lower GMSD is preferred.

### 4.3. Comparison with State-of-the-Art

The proposed CMSP method is compared against four single-frame destriping approaches spanning both classical and learning-based families: D1WLS [5], DLS-NUC [4], DCGAN [6], and ASCNet [7]. All baseline methods are evaluated using their publicly available pretrained weights under the same synthetic noise conditions. VIA-NUC [10], the only other RGB-guided infrared NUC method, is excluded because no public implementation is available and the method was evaluated on a private dataset.

The quantitative results are summarized in Table 2. On the M3FD dataset, where the model is trained and tested, CMSP achieves a PSNR of 51.91 dB, outperforming the strongest baseline DCGAN by 5.79 dB. On the cross-dataset evaluations, where the model is applied without any retraining, CMSP maintains a clear advantage. On MSRS, it achieves 48.57 dB compared to 47.70 dB for DCGAN. On LYNRED, CMSP reaches 46.98 dB, surpassing DCGAN by 2.15 dB. Across all three datasets, CMSP achieves the lowest GMSD and the highest PSNR by a substantial margin. On MSRS, DCGAN retains a marginal SSIM advantage (0.9935 vs. 0.9930), though CMSP outperforms it on both PSNR and GMSD.

### 4.4. Visual Comparison

**Figure 2:**
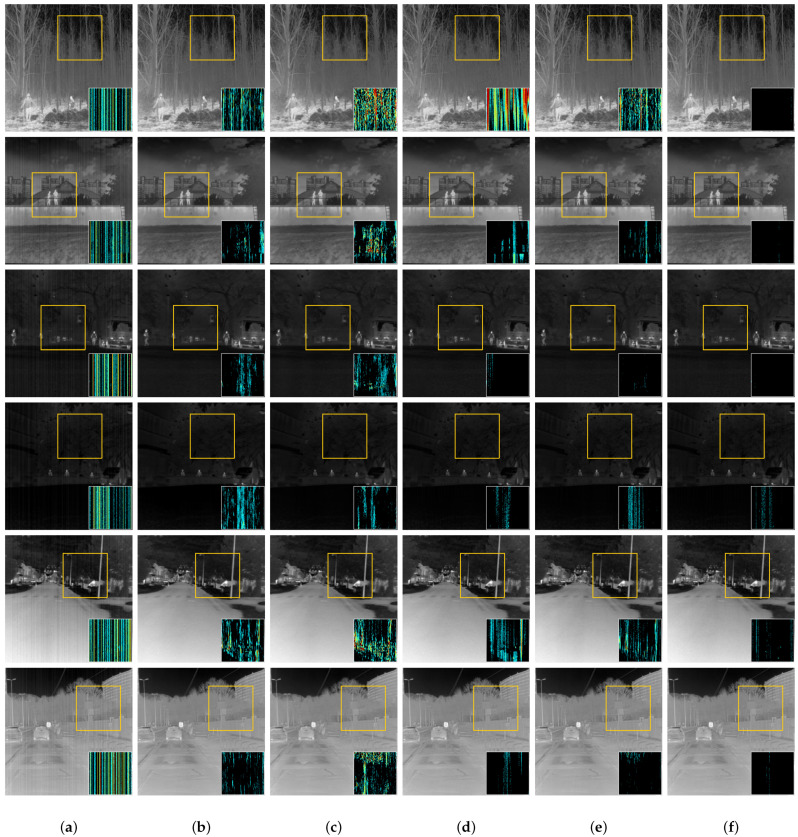
Visual comparison of destriping results on sample images from M3FD (top two rows), MSRS (middle two rows) and LYNRED (bottom two rows). From left to right: (**a**) Noisy IR input, (**b**) ASCNet [7], (**c**) DLS-NUC [4], (**d**) D1WLS [5], (**e**) DCGAN [6], and (**f**) CMSP (ours). The yellow box indicates the cropped region of interest. Insets show the absolute error map (pixel-wise difference from the ground truth) within the yellow-boxed region, displayed with a colormap where darker tones indicate lower error and brighter tones indicate higher error.

Figure 2 presents visual results on representative test images from each dataset, with error maps (absolute difference against ground truth) shown as insets for the cropped region indicated by the yellow box. ASCNet reduces stripe intensity but leaves clearly visible column residuals, and the presence of thick non-columnar artifacts in the error maps suggests that the correction itself introduces additional distortion. DLS-NUC and D1WLS tend to over-smooth vertical scene content along with the stripe noise; this is most apparent in the M3FD tree scene (first row), where tree trunks and branches are visibly blurred and produce strong residuals. DCGAN produces the best results among the baselines, effectively denoising scenes where the other three methods leave high residuals on foreground objects, though faint stripe residuals remain visible in its error maps. The proposed CMSP produces near-zero error maps across all three datasets, with only minor residuals on some images.

### 4.5. Downstream Task Evaluation

To evaluate whether the proposed destriping method benefits downstream applications, we conduct an infrared small target detection (IRSTD) experiment on the IRSTDID-800 benchmark [26]. This dataset contains 500 real-world infrared images with stripe noise and pixel-level small target annotations (IRSTDID-SKY), along with 300 stripe-affected background images without targets (IRSTDID-GND). We apply each destriping method to all 800 images and evaluate detection performance on the destriped outputs. Since the GND subset contains no targets, detections on these images contribute exclusively to the false alarm rate, providing a measure of whether destriping introduces spurious detections in target-free scenes.

Three lightweight detectors and one high-capacity IRSTD detector are selected from the BasicIRSTD (https://github.com/XinyiYing/BasicIRSTD (accessed on 28 May 2026).) toolkit based on parameter count: RDIAN (0.22 M) [27], ACM (0.40 M) [28], ALCNet (0.43 M) [29], and UIU-Net (50.54 M) [30]. All detectors use pretrained weights provided by BasicIRSTD, trained on stripe-free IRSTD-1k [31] dataset. Detection performance is measured by probability of detection ($P_{d}$) and false alarm rate ($F_{a}$) at a fixed threshold of 0.5. Since IRSTDID-800 provides only infrared images without co-registered RGB, CMSP receives a zero-valued RGB input; the AdaptiveSPADE module runs but receives no useful cross-modal guidance. This experiment therefore evaluates CMSP in its infrared-only fallback mode: it assesses the robustness of the architecture as a single-frame infrared denoiser when RGB is unavailable, rather than the effectiveness of cross-modal guidance under real deployment conditions. DLS-NUC is excluded from this experiment due to input size constraints incompatible with the dataset resolution.

Table 3 summarizes the detection results. D1WLS degrades detection performance across all four detectors, reducing $P_{d}$ about 1–3% compared to the no-correction baseline. This is consistent with the over-smoothing of vertical scene content observed in the visual comparison (Section 4.4), which suppresses small target signatures along with stripe noise. DCGAN achieves the highest $P_{d}$ on the three smaller detectors (ACM, ALCNet, RDIAN), but its false alarm rate is consistently elevated, most notably on RDIAN, where $F_{a}$ increases by 61% relative to the no-correction baseline. CMSP provides a favorable balance between detection improvement and false alarm control, improving $P_{d}$ on all four detectors while keeping $F_{a}$ substantially lower than DCGAN and close to the lowest levels achieved by any destriping method. On UIU-Net, the largest detector evaluated (50.54 M parameters), CMSP achieves the lowest $F_{a}$ among all destriping methods. Together with the computational efficiency analysis in Section 4.9, where CMSP uses 47× fewer parameters than DCGAN, these results indicate that CMSP, even when operating without RGB guidance, provides a favorable operating point that preserves small target structures during denoising while keeping spurious detections low.

### 4.6. Ablation Study

To isolate the contributions of the RGB prior branch and the adaptive gating mechanism, we compare three variants of the proposed architecture across all three datasets. CMSP IR-only disables the RGB prior branch entirely, so that AdaptiveSPADE reduces to instance normalization. CMSP Spade enables the RGB prior branch but replaces AdaptiveSPADE with uniform SPADE modulation, applying RGB guidance equally at every spatial location. CMSP Full Model uses the complete architecture with AdaptiveSPADE. All three variants are trained on the same training set and share the same backbone, training configuration, and noise generation pipeline.

As shown in Table 4, uniform SPADE modulation yields a modest improvement in M3FD (+0.85 dB) but provides no benefit on the cross-dataset evaluations. On MSRS, it slightly degrades performance (47.63 vs. 47.66 dB), and on LYNRED, it has no measurable effect. This confirms that applying RGB guidance uniformly can introduce interference where cross-modal correspondence is weak. AdaptiveSPADE resolves this by gating the modulation per pixel, more than doubling the RGB benefit on M3FD (+2.07 dB vs. +0.85 dB) and converting the negative or null effect on MSRS and LYNRED into consistent gains of +0.92 dB and +0.23 dB, respectively. On MSRS, the IR-only variant reaches 47.66 dB, comparable to DCGAN (47.70 dB), and it is specifically the adaptive RGB guidance that pushes the method past the strongest baseline.

In addition to the RGB guidance, we evaluate the contribution of the two branches in the dual-path output head. Starting from the trained model, we disable one branch at a time and re-evaluate on M3FD. With only the residual branch active, PSNR drops to 36.43 dB, whereas with only the column-constant branch active it reaches 48.95 dB, compared to 51.91 dB for the full head. This confirms that both paths contribute in proportion to the structure of the noise: the column-constant branch accounts for the dominant column-structured offset, consistent with the column-parallel readout origin of stripe noise, while the residual branch supplies the smaller scene-dependent correction.

### 4.7. Robustness to Misregistration

To quantify the effect of RGB–IR misregistration on destriping, we evaluate the trained model under controlled spatial perturbations of the guidance input. At test time, the RGB image is shifted diagonally—by an equal offset of {1,2,4,8} pixels in both the horizontal and vertical directions—relative to the infrared image, while the noisy infrared input and the ground truth remain unchanged, and the metrics are recomputed on each dataset.

Table 5 reports PSNR as a function of shift, the degradation is small on all three datasets: an 8-pixel shift reduces PSNR by 0.49 dB on M3FD and by less than 0.2 dB on MSRS and LYNRED. The effect is largest on M3FD, where the aligned RGB margin is also largest, and smallest on LYNRED, where the gate already relies mainly on the infrared input, consistent with a mechanism that weights RGB in proportion to its reliability.

### 4.8. Confidence Map Analysis

**Figure 3:**
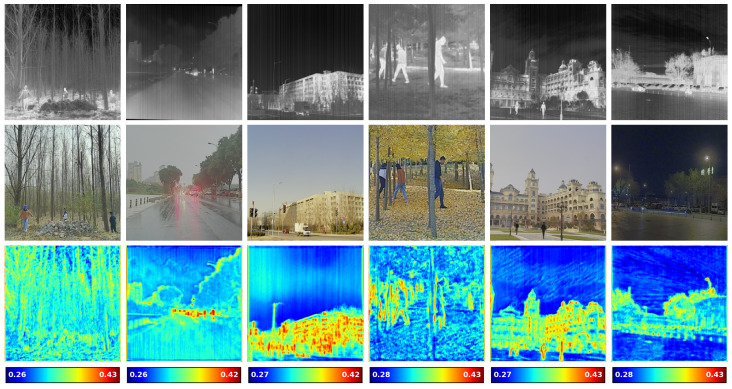
Visualization of the learned confidence map on representative M3FD test images. From top to bottom: Noisy IR image, RGB image, and the confidence map c displayed with the jet colormap. Each map is independently min–max rescaled for display and the raw gate-value range is annotated on the colorbar beneath it.

Figure 3 visualizes the per-pixel confidence map learned by the AdaptiveSPADE module. The gate values vary smoothly across the image rather than saturating to zero or one, indicating that the network learns a continuous weighting between SPADE-modulated and instance-normalized features. Spatially, higher confidence values appear at regions with distinct structural content such as buildings, facades, and object boundaries, where the RGB modality provides informative edge cues. Lower confidence values are observed in uniform regions such as sky and open roads, where instance normalization handles the denoising effectively without external guidance. This spatial pattern aligns with the non-uniform correlation structure identified in Section 3.2 and confirms that the adaptive gating mechanism learns to concentrate RGB guidance where it is most beneficial.

Quantitatively, over the full test split, the mean gate value is close to 0.32 on all three datasets (M3FD 0.322, MSRS 0.323, LYNRED 0.319) with a small standard deviation (0.018–0.022), confirming that the gate operates in a graded regime rather than a binary mask. Binning pixels by clean-IR gradient magnitude, the mean gate increases monotonically from the flattest to the most textured regions on every dataset (e.g., on M3FD from 0.309 to 0.345), confirming that the gate increases RGB reliance where local structure is present.

### 4.9. Computational Complexity

Table 6 reports model size and inference time for each method. CMSP uses 638 K parameters, substantially fewer than ASCNet (4.6 M) and DCGAN (30.4 M). At 31 ms per frame on average, CMSP is also substantially faster than both DCGAN (49 ms) and ASCNet (57 ms), while achieving a 5.79 dB PSNR advantage over the former. This lightweight design makes the proposed architecture suitable for deployment in real-time EO/IR systems where both modalities are available.

## 5. Discussion

The confidence map visualization in Figure 3 confirms that AdaptiveSPADE learns the spatially selective behavior motivated by the correlation analysis. Higher gate values appear at structured regions such as building facades and object boundaries, while lower values appear in uniform areas such as sky. The RGB margin decreases across datasets (2.07 dB on M3FD, 0.92 dB on MSRS, 0.23 dB on LYNRED). The misregistration experiment (Section 4.7) shows this is not driven by RGB–IR alignment: an 8-pixel shift changes PSNR by at most 0.49 dB. We therefore attribute the smaller cross-dataset margins to domain shift between the M3FD training domain and the unseen test domains, with the adaptive gate keeping performance at or above the infrared-only level when guidance is weak.

Two physics-informed design choices contribute to the overall performance of CMSP. The column-mean subtraction applied before confidence estimation removes the dominant column-structured component of the noise analytically, making the gate robust to the stripe noise the network aims to remove, without requiring additional learned parameters. The dual-path output head encodes the column-wise structure of stripe noise through a dedicated branch, reducing the solution space compared to a generic residual estimation approach.

The current formulation has two main limitations. First, CMSP requires co-registered RGB–IR pairs and cannot be used on infrared-only systems. Second, training relies on synthetic stripe noise. While this is standard in the field, as real noisy–clean image pairs are not available, it introduces a potential gap between training and deployment conditions. The downstream experiment on IRSTDID-800 (Section 4.5) shows that the method generalizes to real stripe noise, but a dedicated evaluation on real RGB–IR paired data with native noise is left as future work.

## 6. Conclusions

This paper investigated the use of co-registered visible-light images for single-frame infrared column stripe noise removal. A blockwise correlation analysis revealed that the structural correspondence between RGB and IR horizontal discontinuities is spatially non-uniform, with a notable fraction of image regions exhibiting strong cross-modal agreement while others show negligible correlation. Based on this observation, we introduced AdaptiveSPADE, which uses a learned confidence map to decide where RGB-guided modulation should be applied and where standard instance normalization should be retained. The confidence map is estimated from column-mean-subtracted features so that the gate is less affected by stripe noise itself.

The proposed CMSP architecture, combining stripe-aware depthwise convolutions, AdaptiveSPADE modulation, and a dual-path output head, was evaluated on three public RGB–IR datasets. On M3FD, CMSP achieved 51.91 dB PSNR, outperforming the best baseline by 5.79 dB. Cross-dataset evaluations on MSRS and LYNRED confirmed generalization, with CMSP achieving the highest scores on both datasets without retraining. The adaptive gating mechanism demonstrated the expected behavior of concentrating RGB guidance on structured regions and attenuating it where cross-modal alignment is weak, allowing CMSP to outperform all evaluated baselines while maintaining a compact parameter footprint and practical inference latency.

Future work could extend the approach to full fixed-pattern noise correction by incorporating gain estimation through multi-frame temporal processing. Investigating disparity-robust fusion strategies to improve performance under imperfect RGB–IR registration is another promising direction. Applying the adaptive gating principle to other cross-modal restoration tasks where the guide and target modalities exhibit spatially varying correlation could further broaden the applicability of the proposed framework.

## Figures and Tables

**Table 1:** Blockwise absolute correlation |ρ| between RGB and clean IR column discontinuities on the M3FD dataset. The baseline denotes the expected |ρ| for uncorrelated random signals of the same block size. The tail columns indicate the percentage of blocks exceeding the given correlation threshold.

Block	|ρ|	Baseline	Ratio	>0.2	>0.3	>0.5
8 × 8	0.1871	0.0997	1.9×	34.6%	19.1%	6.6%
16 × 16	0.1429	0.0499	2.9×	22.7%	13.3%	4.7%
32 × 32	0.1239	0.0249	5.0×	20.1%	11.5%	3.9%

**Table 2:** Quantitative comparison on three datasets under synthetic column stripe noise. The best and second best results are highlighted in **bold** and underline, respectively. ↑ indicates higher is better; ↓ indicates lower is better.

Method	M3FD			MSRS			LYNRED		
	PSNR ↑	SSIM ↑	GMSD ↓	PSNR ↑	SSIM ↑	GMSD ↓	PSNR ↑	SSIM ↑	GMSD ↓
ASCNet [7]	38.67	0.9816	0.0090	44.84	0.9855	0.0052	42.24	0.9941	0.0056
DLS-NUC [4]	40.97	0.9847	0.0168	43.39	0.9821	0.0132	41.18	0.9819	0.0149
D1WLS [5]	44.77	0.9962	0.0102	46.25	0.9909	0.0090	42.98	0.9934	0.0130
DCGAN [6]	46.12	0.9964	0.0050	47.70	**0.9935**	0.0039	44.83	0.9948	0.0048
CMSP (Ours)	**51.91**	**0.9994**	**0.0009**	**48.57**	0.9930	**0.0037**	**46.98**	**0.9973**	**0.0030**

**Table 3:** Infrared small target detection results on IRSTDID-800 after applying different destriping methods. $P_{d}$ (↑) is the probability of detection and $F_{a}$ (↓) is the false alarm rate (reported as $F_{a}\times 10^{3}$). Results are reported at a fixed detection threshold of 0.5. The best and second-best results are highlighted in **bold** and underline, respectively.

Condition	ACM		ALCNet		RDIAN		UIU-Net	
	$P_{d}$ ↑	$F_{a}$ ↓	$P_{d}$ ↑	$F_{a}$ ↓	$P_{d}$ ↑	$F_{a}$ ↓	$P_{d}$ ↑	$F_{a}$ ↓
No Correction	0.914	0.248	0.904	0.213	0.839	0.193	0.900	0.093
ASCNet [7]	0.933	0.245	0.913	0.238	0.871	0.278	**0.918**	0.113
D1WLS [5]	0.899	**0.232**	0.875	**0.208**	0.830	0.246	0.877	0.101
DCGAN [6]	**0.939**	0.250	**0.924**	0.242	**0.881**	0.310	0.913	0.101
CMSP (Ours)	0.932	0.238	0.913	0.214	0.868	**0.239**	0.915	**0.101**

**Table 4:** Ablation study: effect of RGB guidance and AdaptiveSPADE blocks on PSNR (dB) across three datasets. The RGB margin denotes the improvement provided by the visible-light input.

Configuration	M3FD	MSRS	LYNRED
CMSP IR-only	49.84	47.66	46.75
CMSP Spade	50.69	47.63	46.75
CMSP Full Model	51.91	48.57	46.98
RGB margin	+2.07	+0.92	+0.23

**Table 5:** Effect of RGB–IR misregistration on PSNR (dB). The RGB input is shifted diagonally by the indicated number of pixels at test time, with the infrared input and ground truth unchanged.

RGB Shift (px)	M3FD	MSRS	LYNRED
0 (aligned)	51.91	48.57	46.98
1	51.86	48.57	46.93
2	51.79	48.55	46.90
4	51.62	48.53	46.82
8	51.41	48.50	46.81

**Table 6:** Model complexity comparison for unified 288×288 test images. Inference time is averaged over the three test datasets M3FD, MSRS, and LYNRED.

Method	Params (K)	GFLOPs	Time (ms)
D1WLS ^†^ [5]	–	–	1059±48
DLS-NUC [4]	84	1.67	23.1±6.1
CMSP (Ours)	638	62.08	30.5±6.3
ASCNet [7]	4642	47.78	56.5±4.8
DCGAN [6]	30,397	73.78	48.6±5.4

^†^ Classical optimization method executed on CPU; all others use GPU inference.

## Data Availability

The datasets used in this study are publicly available. M3FD is available at https://github.com/JinyuanLiu-CV/TarDAL (accessed on 28 May 2026). MSRS is available at https://github.com/Linfeng-Tang/MSRS (accessed on 28 May 2026). The Lynred Mobility Dataset is available at https://www.lynred.com/lynred-mobility-dataset (accessed on 28 May 2026). The source code of the proposed method is available at https://github.com/cescript/cmsp (accessed on 28 May 2026).
